# In Vitro Selection of DNA Aptamers that Binds Geniposide

**DOI:** 10.3390/molecules22030383

**Published:** 2017-02-28

**Authors:** Aozhe Zhang, Dingran Chang, Zijian Zhang, Fan Li, Weihong Li, Xu Wang, Yingfu Li, Qian Hua

**Affiliations:** 1 School of Basic Medical Science, Beijing University of Chinese Medicine, 11 East Road, North 3rd Ring Road, Chaoyang District, Beijing 100029, China; aozhezhang@outlook.com (A.Z.); liweihong.403@163.com (W.L.); wangxubucm@sina.com (X.W.); 2 Department of Biochemistry and Biomedical Sciences, McMaster University, 1280 Main Street West, Hamilton, ON L8S 4K1, Canada; changd3@mcmaster.ca (D.C.); lifan@mcmaster.ca (F.L.); liying@mcmaster.ca (Y.L.); 3 Beijing Institute of Traditional Chinese Medicine, Beijing University of Chinese Medicine, 11 East Road, North 3rd Ring Road, Chaoyang District, Beijing 100029, China; zhangzijianQQ1012@163.com

**Keywords:** geniposide, aptamer, in vitro selection, DNA, Chinese herbal medicine, molecular recognition

## Abstract

Geniposide is a key iridoid glycoside from *Gardenia jasminoides* fructus widely used in traditional Chinese herbal medicine. However, detection of this small molecule represents a significant challenge mostly due to the lack of specific molecular recognition elements. In this study, we have performed in vitro selection experiments to isolate DNA aptamers that can specifically bind geniposide. Using a stringent selection procedure, we have isolated DNA aptamers that can distinguish geniposide from genipin and glucose, two structural analogs of geniposide. Two top aptamers exhibit low micromolar binding affinity towards geniposide, but show significantly reduced affinity to genipin and glucose. These aptamers have the potential to be further developed into analytical tools for the detection of geniposide.

## 1. Introduction

Chinese herbal medicine (CHM) has been practiced for several thousand years in Asia and has gained increasing popularity in the West both as dietary supplements and alternative medicine [1,2]. It has been reported that the use of herbal medicines in China accounts for 40% of all health care services, and 38%–75% of people in the West has used herbal medicines at least once [3,4]. This is not surprising given the fact that a large number of scientific studies over the past decades have shown that the herbal materials contain natural compounds that are pharmaceutically valuable [5]. 

**Figure 1:**
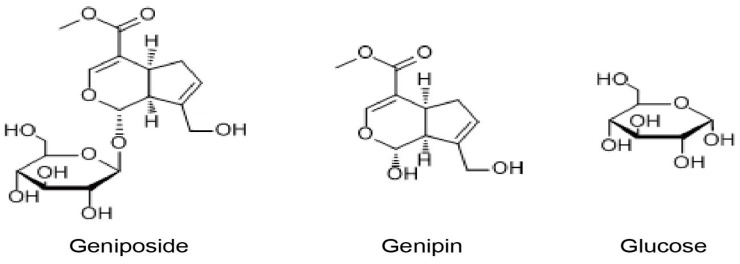
Chemical structures of geniposide, genipin and glucose.

Geniposide (Figure 1) is the key medical ingredient of the Fructus Gardeniae which is widely used as a CHM [6]. As an iridoid glycoside compound, geniposide is unstable, non-volatile and thermally labile polar [7]. It has been an ideal raw material for natural pigment and natural crosslinking reagent for biological tissue fixation [8,9]. During the past years, there was an increasing interest in studying the potent application valuable of geniposide. It has been shown in many studies to confer notable medical benefits, such as antioxidation, anti-inflammatory, anti-atherosclerosis, anti-ischemic brain injuries, anti-platelet aggregation, anti-hyperglycemia, anti-hypertension [10,11,12,13,14,15,16,17,18]. Besides the hepatotoxicity of geniposide has been demonstrated in rats, geniposide is also an active ingredient of Qingkailing injection which was reported to cause serious adverse reactions [19,20,21]. However, the precise mechanisms of its various effects remain poorly understood.

Increasing popularity of geniposide also brings in safety concerns [3]. Misidentification and counterfeiting products that do not contain the listed ingredients represent common problems [22,23,24]. The use of unqualified, false and inferior geniposide product as medicinal ingredients and dietary supplements could cause serious side effects and is unsafe for people. One important strategy to deal with these problems is to develop simple but effective analytical methods that can conveniently detect geniposide. However, geniposide is a small molecule, like most pharmacologically active compounds in CHM, and its detection and monitoring require the use of complicated analytical instruments such as high-performance liquid chromatography (HPLC). These techniques cannot be used for self-monitoring by patients themselves. Therefore, there is a growing interest in developing simpler, portable sensors for such applications. Development of portable sensors needs effective molecular recognition elements (MREs). However, engineering MREs for small molecules represents a significant challenge simply because it is extremely difficult to raise antibodies to small molecules due to their poor immunogenicity. The key objective of this study is to examine the possibility of deriving DNA aptamers—MREs made of synthetic DNA molecules—for geniposide, a key bioactive ingredient from CHM. 

Aptamers are single-stranded DNA or RNA molecules capable of binding to a specific target. They are isolated in vitro through a process known as the Systematic Evolution of Ligands by Exponential enrichment (SELEX), a technique originally reported in 1990 [25,26]. Since then, a large number of aptamers have been discovered for a wide range of targets that include metal ions, biological cofactors, proteins and even cells [27,28,29]. Aptamers have analogous function of antibodies but also offer distinct advantages that include smaller size, higher stability, lower cost and better target inclusiveness. For these reasons, aptamers have become a popular choice of MRE for the development of next-generation biosensors [29,30,31,32]. 

We sought to develop DNA aptamers that can specifically recognize geniposide, which is made of genipin and glucose moieties. Since it can be hydrolyzed to form a glucose and an aglycone genipin, we employed a counter-selection approach to search for specific aptamer sequences that have high affinity for geniposide as a whole but do not bind strongly to either genipin or glucose (Figure 1).

## 2. Results

### 2.1. SELEX Strategy

**Figure 2:**
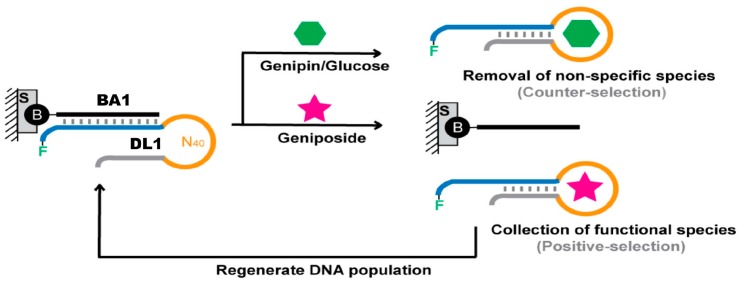
SELEX (Systematic Evolution of Ligands by Exponential Enrichment) strategy. The DNA library (DL1) is hybridized with a biotinylated antisense DNA (BA1) immobilized on a streptavidin (S) based affinity column. DNA molecules with cross-activity to genipin and glucose are removed through washes with a solution containing these two counter-selection targets. The desired binding sequences are then eluted with a solution containing geniposide. These sequences are amplified and used for the next round of selection. B: biotin; BA1: biotinylated antisense DNA; N_40_: 40 random nucleotides; F: fluorescein.

We employed a SELEX strategy that has been proven successful for selecting aptamers for small-molecule targets [33,34,35,36]. The method was based on the concept of structure-switching aptamers [37,38]. Since a DNA aptamer is a single-stranded DNA molecule, it can form a Watson-Crick duplex structure with a complementary DNA sequence. However, when the target for the aptamer is present, the aptamer will switch from the duplex structure to the aptamer/target complex structure. To select aptamers by this structure-switching mechanism, one simply needs to immobilize the DNA pool onto a solid support that contains the antisense sequence that can hybridize to the DNA molecules in the DNA pool and elute potential DNA aptamers with the target of interest, as depicted in Figure 2. 

All the DNA molecules used for the SELEX experiment are provided in Table 1. DL1 is the DNA library, which contains 40 random nucleotides in the middle portion of the sequence and two fixed-sequence flanks that serve as primer-binding sites for DNA amplification via polymerase chain reaction (PCR). The 5′-fixed sequence also functions as the hybridization tag to create an affinity column and thus is designed to bind BA1, a biotinylated DNA molecule with a complementary sequence to the 5′-domain of DL1. Therefore, mixing DL1, BA1 and streptavidin-modified agarose beads will lead to the immobilization of DL1 onto agarose beads, creating a separation mechanism to enable aptamer selection. 

There are six key steps in our SELEX strategy. Following step 1 in which the DNA library is immobilized on agarose beads, counter-selection targets are used in step 2 to eliminate DNA molecules that bind either glucose or genipin alone. This is followed by step 3 where geniposide is used as the positive selection target. In this step, the desired aptamers should make a transition from the duplex state to the DNA/geniposide complex state and release themselves from the column to the solution. These molecules are collected and amplified by two PCR steps. The first PCR step (step 4), conducted with the use of FP1 and RP1 as the primer set, simply serves as the amplification step to produce copies of the aptamer sequences. The second PCR step (step 5) is designed to facilitate the production of fluorescently labeled single-stranded aptamer candidates to be used for the next round of selection. For this purpose, a modified set of primers—FP2 and RP2—are used. FP2 has the identical sequence of FP1 but it has a fluorescein tag at the 5′-end to label the aptamer strand. RP2 is equivalent to RP1, but it contains a hexaethylene glycol spacer (18 atoms) and T_15_ head at the 5′ end. The spacer prevents the polyT sequence from being amplified, making the non-aptamer strand 15 nucleotides longer than the aptamer strand so that the aptamer candidates can be simply purified via 10 % denaturing (8 M urea) polyacrylamide gel electrophoresis (dPAGE) in step 6. The recovered aptamer candidates are then reannealed to BD1 on agarose beads to begin the next round of selection. 

### 2.2. The SELEX Progress

**Figure 3:**
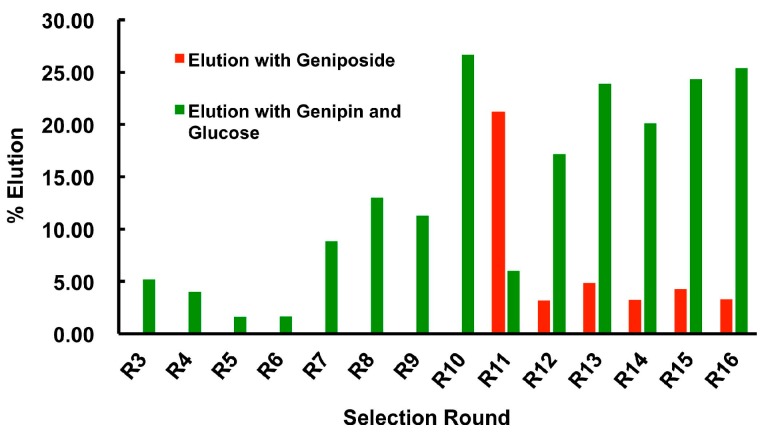
SELEX progress. % Elution was calculated as fluorescence of target-eluted DNA divided by the total fluorescence of column-bound DNA. R: round of selection.

A total of 16 rounds of selection were carried out and counter-selection was not performed until round 11 (Figure 3). We monitored the progress of selection from round 3 by measuring the fluorescence intensity of geniposide-eluted DNA fraction, normalized by the total fluorescence of bead-bound library. Without a counter-selection step, approximately 25% of the DNA library was eluted in the positive selection step by round 10. However, the enriched DNA pool did not exhibit high specificity for geniposide, revealed by the observation ~20% column-bound DNA were eluted by the mixture of genipin and glucose. At this point, a counter-selection step was introduced. By round 16, the DNA pool showed a significant improvement in geniposide-dependent elution: geniposide was able to elude ~25% of column-bound DNA while the mixture of genipin and glucose produced about 3% elution. The pool from round 16 was then subjected to deep sequencing followed a previously published protocol [39,40,41]. Top 6 sequence classes are listed in Table 2 (see details of deep sequencing result in Supplementary Materials Table S1).

### 2.3. Putative Aptamer Structures

**Figure 4:**
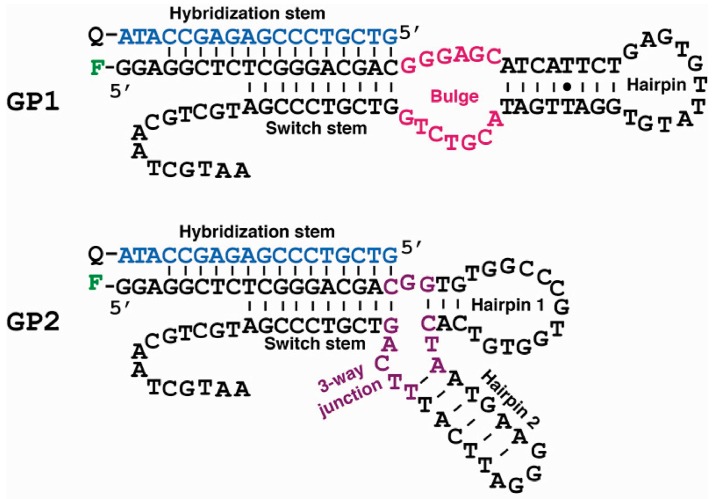
Predicted secondary structures of GP1 and GP2, the top 2 aptamers from the selection. GP1 had an internal bulge element, highlighted in pink, which was suspected to be the binding site for geniposide. GP2 contained a 3-way junction motif, highlighted in purple, which was suspected to be the binding site for geniposide. Each aptamer was labeled with a fluorescein molecule at the 5′-end for the experiments to determine the binding affinity and specificity. The blue strand was the complementary DNA (cDNA), which was labeled with a dabcyl quencher (Q) for the binding affinity and specificity experiments.

The top 2 classes, named GP1 and GP2, were chosen for secondary structure analysis (Figure 4), through the use of the mfold Web Server for single-stranded DNA structure prediction [42]. Both aptamers contained a switch stem that was purposely designed to facilitate target-induced competition with the hybridization stem (which was used to immobilize the library onto beads). Interestingly, the predicted structure of GP1 contained a hairpin that flanked an internal bulge consisted of 13 nucleotides (shown in pink). We speculated that the bulge element acted as the binding site for geniposide. GP2 exhibited a more complex structure: it had two hairpin elements and a 3-way junction motif (shown in purple) created by the three internal stems. We speculated that the 3-way junction element may act as the binding site for geniposide.

### 2.4. Binding Affinity

**Figure 5:**
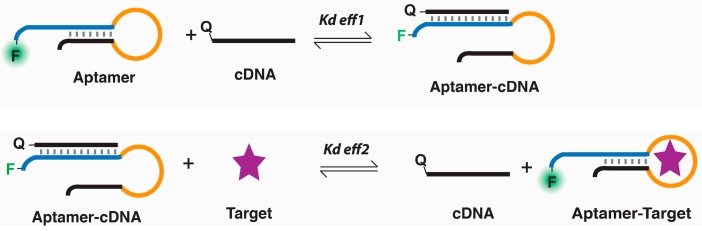
Graphic illustration of reactions for determining the binding affinity of a structure-switching aptamer for its target. *K*_d:_ dissociation constant.

To determine the dissociation constants (*K*_d_) of GP1 and GP2 against geniposide, we used a method illustrated in Figure 5, which was originally developed by Hu and Easley [43]. There were two separate binding affinity measurements for each aptamer, *K*_d,eff1_ and *K*_d,eff2_. *K*_d,eff1_ is the dissociation constant between the aptamer and capture DNA sequence (named complementary DNA (cDNA)), which can be calculated by Equation (1):

*K*_d,eff1_ = [aptamer][cDNA]/[aptamer-cDNA] (1)

For this purpose, the aptamer was labeled with a fluorescein and cDNA was labeled with the dabcyl quencher. When the aptamer (used at 50 nM) was mixed with cDNA (varied between 0 and 500 nM), the fluorescence intensity of the aptamer solution would decrease. *K*_d,eff2_ is a unitless constant measuring the equilibrium of target-induced structure switching process, which can be calculated by Equation (2):

*K*_d,eff2_ = [cDNA][aptamer-target]/[aptamer-cDNA][target] (2)

*K*_d_ can then be calculated using Equation (3):

*K*_d_ = *K*_d,eff1_/*K*_d,eff2_ (3)

**Figure 6:**
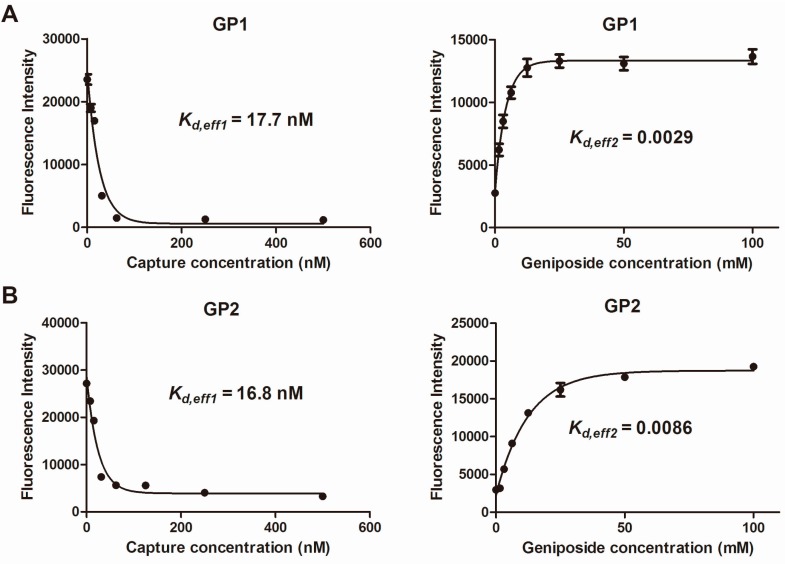
Experimental data for deriving *K*_d_ for GP1 (**A**) and GP2 (**B**). Capture refers to cDNA. *K*_d,eff__1_ of GP1 was ~17.7 nM and GP2 was ~16.8 nM; and *K*_d,eff2_ of GP1 was ~2.9 × 10^−3^ and GP2 was ~8.6 × 10^−3^, respectively. Using equation: *K*_d_ = *K*_d,eff2_/*K*_d,eff2_, *K*_d_ of GP1 was ~6.1 μM and GP2 was ~2.0 μM.

The data for measuring relevant constants for GP1 and GP2 were provided in Figure 6. Using this method, we estimated that the *K*_d_ values of GP1 and GP2 against geniposide were 6.1 μM and 2.0 μM, respectively.

### 2.5. Recognition Specificity

**Figure 7:**
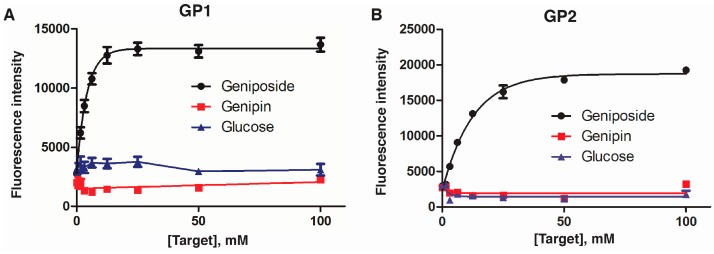
Experimental data for the recognition specificity of GP1 (**A**) and GP2 (**B**) for its targets—geniposide (black), genipin (red) and glucose (blue).

We also performed experiments to compare the relative affinities of GP1 and GP2 towards geniposide, genipin and glucose and the data were provided in (Figure 7). Three targets were added to the aptamer-cDNA solutions and their fluorescence responses were monitored. We found that both glucose and genipin were unable to cause detectable switch of GP1 and GP2 from the cDNA/aptamer duplex state to aptamer/target complex state, which prevented us from obtaining a *K*_d,eff2_ value to derive a disassociation constant. These results indicated that these two aptamers were highly specific for geniposide. 

## 3. Discussion

In this work, we have isolated several DNA aptamers that bind geniposide, a major medical component of Fructus Gardeniae and examined the binding affinity and specificity of the top two aptamers, GP1 and GP2. Both aptamers exhibit a low micromolar binding affinity towards geniposide but do not show detectable affinity towards genipin and glucose under similar binding conditions. The structure-switching strategy was chosen to set up SELEX for aptamer selection mainly for the fact that this method does not require the creation of affinity columns, which can be difficult for small-molecule targets [44,45]. Most of the bioactivity ingredients in CHM are small molecules [46], which typically do not have functional groups that are suitable for immobilization on solid surfaces. Compared to the conventional SELEX, structure-switching strategy not only has the advantages of avoiding immobilization of targets but also minimizing the isolation of non-specific aptamers, given the fact that in classic SELEX, the selection is being performed on a target-matrix conjugate rather than the target alone. Under these circumstances, non-specific binding may take place and may affect future applications when the target is free in solution. A random 40 nt sequence was used instead of 30 nt [34,36], to increase the possibility to generate high affinity aptamers, however, it means performing more rounds and consuming more time. Fluorescein labeling was very convenient for monitoring and avoided radioactive labeling. In addition, fluorescent labeling introduced from the beginning would not affect the binding affinity with the target molecules for further use concern.

To achieve high specificity, two constituents were employed in the counter-selection. Because geniposide are made of genipin and glucose moieties, we used genipin and glucose as counter targets to remove sequences binding to the compound with a single moiety (genipin or glucose). Only the sequences binding to geniposide as a whole could be selected in our strategy. The major components of Gardenia fruits are iridoid glycosides including geniposide, gardenoside, genipin-1-*O*-β-gentiobioside, geniposidic acid, and scandoside methyl ester [47]. These iridoid glycosides are the analogues of geniposide and they are likely to be present in CHM [48]. Such analogues have a common feature that they have either a genipin or a glucose moiety. As these analogues do not contain both of the two moieties, the developed aptamers are likely to have a high specificity for geniposide against the other iridoid glycosides. Although it remains to be further verified in subsequent experiments, the idea of introducing constituents as counter targets was innovative compared to using analogues directly. Additionally, it would reduce expenses by avoiding every analogue which is of high price. 

To our knowledge, our work represents the first attempt of applying SELEX to create DNA aptamers as MREs for key bioactive ingredients from CHM. It is conceivable that the same strategy can be adopted for creating novel aptamers for other small-molecule ingredients in CHM.

As the popularity of herbal medicines rising worldwide, safety issues have become more important. The use of geniposide as medicinal ingredients and dietary supplements comes with safety and quality concerns [49]. Our inability to reliably distinguish geniposide from their close relatives, inferior substitutes, adulterants, and counterfeits represents a significant challenge in protecting the safety of users and herb efficacy. Combating these issues need effective methods for the detection of geniposide that are suitable for on-demand testing by consumers. Current technologies, which include morphological, microscopic, chemical identification and DNA barcoding, have played a pivotal role in herb drug authentication and quality control [50]; however, these methods are not compatible with self-monitoring applications because they use tests that have to be conducted at centralized laboratories by trained experts using expensive equipment [51]. Compared to antigeniposide antiserum [52] and liquid chromatography-mass spectrometry based methods [53], DNA aptamers have the advantage of being used as simple MREs to set up simple methods to detect geniposide. With high-quality aptamer in hand, we hope to develop effective assays in the near future that can significantly simplify geniposide detection.

## 4. Materials and Methods

### 4.1. DNA Oligonucleotides and Chemical Reagents

All DNA oligonucleotides were purchased from Integrated DNA Technologies (IDT, Coralville, IA, USA). Unmodified DNA oligonucleotides were purified by 10% preparative denatured (8 M urea) polyacrylamide gel electrophoresis (dPAGE), followed by elution and ethanol precipitation. Modified oligonucleotides were purified by reverse-phase HPLC. Purified oligonucleotides were dissolved in water and their concentrations were determined spectroscopically. Geniposide and genipin were purchased from Bioway Biological Technology (Beijing, China). Water used in all experiments was deionized distilled water. All other compounds were purchased from Sigma-Aldrich (Oakville, ON, Canada) unless otherwise noted.

### 4.2. Stock Solutions for SELEX

The following stock solutions were prepared and used for the SELEX experiment: 1× SB (SELEX buffer): 20 mM HEPES, 0.5 M NaCl, 10 mM MgCl_2_, 5 mM KCl, pH 7.4). It was prepared as a 2× stock. Stocks of geniposide, genipin and glucose: a stock solution at 100 mM was made for each of these compounds by dissolving each compound in water. These solutions were stored at 4 °C. 

### 4.3. In Vitro Selection Procedure

The streptavidin-agarose affinity resin was prepared with 250 μL of the streptavidin-agarose resin (binding capacity: 15–28 μg biotin/mL of settled resin; Thermo Scientific, Ottawa, ON, Canada) in a micro Biospin chromatography column (BioRad, Mississauga, ON, Canada). To equilibrate the column, the streptavidin-agarose resin was washed 10 times with 250 μL of 1× SB. 

For the first round of SELEX, a mixture containing 600 pmoles of BD1 and 150 pmoles of DL1 was prepared in 250 μL of 1× SB and incubated at 95 °C for 5 min. The mixture was cooled down to room temperature for 10 min, then passed through a streptavidin agarose column three times. The column was washed ten times with 1× SB. This was followed by elution twice with 250 μL of 1 mM geniposide in 1× SB with incubation at room temperature for 30 min. The two eluents were combined, and the DNA in the solution was concentrated by ethanol precipitation, and dissolved in 50 μL of water. 

The DNA sample obtained above was then subjected to DNA amplification in two consecutive polymerase chain reaction (PCR), first using FP1 and RP1 as the primer set and then FP2 and RP2 as the primer set. The detailed protocols are provided below:

PCR1: eight PCR reactions (50 μL each) were performed. Each reaction mixture was made of 2.5 μL of FP1 (10 μM), 2.5 μL of RP1 (10 μM), 33.5 μL of H_2_O, 5 μL of 10× PCR buffer, 5 μL of the dNTP (dATP, dCTP, dGTP and dTTP) mix (2 mM each dNTP), and 0.5 μL of DNA polymerase (2.5 units) and 1 μL of the eluted DNA product. PCR is carried out under the following condition: 1 cycle of 95 °C (5 min), N cycle(s) of 95 °C (45 s), 60 °C (45 s), 72 °C (45 s), and 1 cycle of 72 °C (10 min). The number of PCR cycles was determined by running reaction mixtures taken at different cycles on a 3% agarose gel.

PCR2: 42 PCR reaction mixtures (50 μL each) were performed. Each reaction mixture was set up similarly with the following changes: FP2 to replace FP1, RP2 to replace RP1, 1 μL of PCR1 amplified DNA as the template. PCR conditions were identical to PCR1.

All 42 PCR2 mixtures were then combined and the amplified DNA was concentrated by ethanol precipitation. The aptamer coding strand was then purified by 10% denatured PAGE. Note that RP2 contains a 15-nucleotide overhang at the 5′ end separated by a triethylene glycol linker, which cannot be amplified by DNA polymerase. This makes the non-DNAzyme-coding strand 15-nucleotide longer than the aptamer strand. 

Gel-purified DNA was then dissolved in 50 μL of water and its concentration was determined spectroscopically. 150 pmol of thus purified DNA was used for the next round of selection.

### 4.4. Binding Affinity Measurement

For the experiment to derive *K*_d,eff1_, the concentration of fluorescein-labeled aptamer was set at 50 nM fixed, while the concentration of dabcyl-labeled cDNA was varied between 0 and 500 nM range. An equal volume of 2× concentrated aptamer and cDNA, both in 1× SB, were mixed, treated at 95 °C for 5 min, and cooled down slowly (~30 min) to room temperature (~23 °C). Each mixture was transferred into a well in a 96-well microplate (non-binding surface, flat bottom; black polystyrene assay plates; Corning, Corning, NY, USA). The fluorescence intensity of each well was recorded on a Tecan Infinite M1000 PRO microplate reader (Tecan, Männedorf, Switzerland). The fluorescence measure was done with a 490 nm excitation filter and a 520 nm emission filter. The fluorescence intensity readings at varying concentrations of cDNA were then fitted through nonlinear least-squares progression using GraphPad Prism 5.0 (GraphPad Software, CA, USA) to generate *K*_d,eff1_. 

For the experiment to derive *K*_d,eff__2_, the final concentration of both the aptamer and the cDNA was kept at 50 nM while the concentration of geniposide was varied between 0 and 100 mM. The aptamer and the cDNA were mixed in 1× SB, and the mixture was then heated at 95 °C for 5 min. Following cooling down at room temperature for 30 min, an equal volume of geniposide (also in SB) was added, and incubated at room temperature for 60 min. Each mixture was then transferred into a microplate well and its fluorescence intensity was recorded. All measurements were made in at least triplicate. The fluorescence intensity readings were then plotted using nonlinear least-squares fitting using GraphPad Prism 5.0. 

### 4.5. Recognization Specificity Measasurement

This method was the same with what we did in the experiment to derive *K*_d,eff__2 _ for geniposide. Prepare a range of 0–200 mM concentration solutions of geniposide, genipin and glucose in 1× SB. The fluorescein-labeled aptamer and dabcyl-labeled cDNA (both the concentration of the aptamer and the cDNA were 100 nM) were mixed and incubate at 95 °C for 5 min, and cool down at room temperature for 30 min. Add an equal volume of target solutions to the above mixture and incubate for 60 min. Then the fluorescence was measured with a 490 nm excitation filter and a 520 nm emission filter on the Tecan microplate reader (Tecan, Männedorf, Switzerland). The binding curves are plotted using nonlinear least-squares fitting using GraphPad Prism 5.0. 

## 5. Conclusions

In summary, we have succeeded in generating aptamers against geniposide with high affinity and specificity. The dissociation constants of the top two aptamers were 6.1 μM and 2.0 μM. To our knowledge, this is the first study applying DNA aptamers for geniposide, a key bioactive ingredient from CHM. This study has further verified that structure-switching selection is an ideal selection procedure to generate aptamers for small molecules including the ingredients from CHM. With the development of these high-quality aptamers, we hope to develop effective assays in the near future that can significantly simplify geniposide detection.

## Figures and Tables

**Table 1:** Sequences of all the DNA molecules used for the SELEX experiment.

Name	Sequence
DL1	5′-GGAGGCTCTCGGGACGAC-N_40_-GTCGTCCCGATGCTGCAATCGTAA-3′
BD1	5′-GTCGTCCCGAGAGCCATA-Biotin-3′
FP1	5′-GGAGGCTCTCGGGACGAC-3′
FP2	5′-Fluorescein-GGAGGCTCTCGGGACGAC-3′
RP1	5′-TTACGATTGCAGCATCGGGACG-3′
RP2	5′-T_15_-X_18_-TTACGATTGCAGCATCGGGACG-3′ ^1^

^1^ This sequence contains a X_18_ spacer (hexaethyleneglycol; 18 denotes 18 atoms, 12 carbon atoms and 6 oxygen atoms) made of that separates the polyT head from the rest of the sequence.

**Table 2:** Sequences of top 6 aptamers.

Name	Sequence ^1^	Number of Copies
GP1	GGGAGCATCATTCTGAGTGTTATGTGGATTGATACGTCTG	934
GP2	GGTGTGGCCCGTGGTGTCACTAATGAAGGGATTCATTTCA	713
GP3	GGCAGCCGATGTTCAATATATTAGTGACTCAGCGACTACT	252
GP4	GGCACATGACCGTTGATTTTCGCAGTTCCGTCATGCGATG	233
GP5	GGGGGCATCTAGCGCGCATTATAGCACACTGGTTGTTCAT	220
GP6	TCACGTACTGGATATACAGCCTGAGGATCCTGAGGTGGTA	200

^1^ Each sequence is written in a 5′ to 3′ direction; only the random portion of each aptamer was shown; each aptamer also contained GGAGGCTCTCGGGACGAC at the 5′ end and GTCGTCCCGATGCTGCAATCGTAA at the 3′ end.

## Supplementary Materials

Supplementary materials are available online. Table S1: List of top 20 sequences from the selection process.
