# Supplementary material for: In Vitro Selection of DNA Aptamers that Binds Geniposide
Source: Molecules. 2017 Feb 28;22(3):383. doi: 10.3390/molecules22030383 (PMC6155188; doi:10.3390/molecules22030383)
Supplement: Supplementary file 1 [file molecules-22-00383-s001.pdf]

Supplementary Materials

Table S1. List of top 20 sequences from the selection process.

Class	Random Sequence [1] N_{40}	Copies
GP 1	GGGAGCATCATTCTGAGTGTTATGTGGATTGATACGTCTG	934
GP 2	GGTGTGGCCCGTGGTGTCACTAATGAAGGGATTCATTTCA	713
GP 3	GGCAGCCGATGTTCAATATATTAGTGACTCAGCGACTACT	252
GP 4	GGCACATGACCGTTGATTTTCGCAGTTCCGTCATGCGATG	233
GP 5	GGGGGCATCTAGCGCGCATTATAGCACACTGGTTGTTCAT	220
GP 6	TCACGTACTGGATATACAGCCTGAGGATCCTGAGGTGGTA	200
GP 7	GGGAGCATCCGTATGTGGTCTAAGTCCGTGGGGTACTCTT	177
GP 8	GGGAGCAGTGTTCAAACCGAGTAGAATTTGTCACCATCTT	177
GP 9	GGCACGCAATATGCACTCTCTGAGTCCTATAGTACGGACT	162
GP 10	GGCACCAATGAAGTGTATATCATATCGGCTATTGGCGTAA	147
GP 11	GGCACCGAAAACATGTGCTAGTCTAATGTTGAGGCGTTAA	146
GP 12	GGGCCGACCAATATTAAGTGTTGTTAATTTGGCCAGATCT	141
GP 13	GGCGCAGTCTTGATGATCTCTAGACCATAGATACTCCGAT	137
GP 14	GGGAGCACTCAATTCGACAAAGAGGTATTTGAGCGTTCAA	132
GP 15	GGGAGCCAAGGGTGAGTATCTATCATCTCAACGTCCTCAT	131
GP 16	GGGAGCACAATAGGTATGTCACATCATAGTATTGCCTTCT	123
GP 17	GGCAGCAAACTTTCTAGGCACAGTTGAAGGTTCGTCTGAT	120
GP 18	GCAGGCACAGAAAGAGATTACAATCCGATACTGCGTCCTA	120
GP 19	GGGATCCATTTGCAGAGATATTTGTCTGGCTTTGGCTCAT	118
GP 20	GGGCAGTGCAGTTCTAACACTATGCTAGTACCAGCTACTT	118

[1] Each sequence is written in a 5' to 3' direction; only the random portion of each aptamer was shown; each aptamer also contained GGAGGCTCTCGGGACGAC at the 5′ end and GTCGTCCCGATGCTGCAATCGTAA at the 3′ end.
